# Exploring the Relationship Between Biochar Pore Structure and Microbial Community Composition in Promoting Tobacco Growth

**DOI:** 10.3390/plants13212952

**Published:** 2024-10-22

**Authors:** Linyuan Yang, Shichen Li, Waqar Ahmed, Tao Jiang, Fupeng Mei, Xiaodong Hu, Wubo Liu, Fatima M. Abbas, Rujun Xue, Xiaoci Peng, Zhengxiong Zhao

**Affiliations:** 1 Yunnan Agricultural University, Kunming 650201, China; 2 Yunnan Academy of Agricultural Sciences, Institute of Tropical and Subtropical Cash Crops, Baoshan 678000, China; 3 Department of Biology, Faculty of Sciences and Arts, King Khalid University, Dahran Al-Janoub 61421, Saudi Arabia

**Keywords:** biochar, pore structure, plant growth promotion, soil microbial communities, pore structure, plant–microbe interactions

## Abstract

The potential benefits of biochar, a carbon-rich substance derived from biomass, for enhancing agricultural yield and soil health have drawn increasing interest. Nevertheless, owing to the lack of specialized studies, the role of its poly-spatial structure in the success of fostering plant growth remains unclear. This study aimed to assess the effects of various biochar pore shapes on tobacco growth and the underlying microbiological processes. Three pyrolysis temperatures (250 °C, 400 °C, and 550 °C) were used to produce biochar from tobacco stems, resulting in different pore structures (T3 > T2 > T1). We then used BET-specific surface area (BET), t.Plot micropore specific surface area (t.Plot), mesopore specific surface area (MSSA), specific pore volume (SPV), average pore size (AP), and mesopore pore volume (MPV) measurements to evaluate the effects of these biochars on tobacco growth and biomass accumulation, and microbial analyses were performed to investigate the underlying mechanisms. When applied to plants, biochar increased their growth compared to untreated controls. The most notable improvement in tobacco growth was observed in the biochar produced at 400 °C (T3), which possessed the largest and most advantageous pore structure among all treatments. Further studies demonstrated that biochars with greater specific surface areas (BET, t.Plot, and MSSA) positively altered the abundance of key microbial taxa (e.g., *Stenotrophobacter*, *Ensifer*, *Claroideoglomus*) and community composition, thereby encouraging plant development and biomass accumulation. Conversely, greater pore volumes (SPV, AP, and MPV) inhibited microbial activity and significantly affected growth and biomass accumulation. Structural equation modeling further demonstrated that the pore structure of biochar greatly affected plant growth by changing the relative abundance and community composition of soil microbes. Maximizing the benefits of biochar in stimulating plant growth and improving soil microbial communities depends on optimizing the material’s pore structure, particularly by increasing the specific surface area. These findings will help expand the use of biochar in sustainable agriculture.

## 1. Introduction

Biochar is produced by the thermal transformation of organic matter in an oxygen-limited environment [1,2]. This carbon-rich material can be obtained from various biomass sources, such as woody materials, livestock manure, agricultural straw, and organic wastes, including municipal biosolids and sewage sludge [3,4]. Because biochar can potentially improve soil health and agricultural yield, its application has attracted increasing attention in recent years. Further, biochar can enhance plant growth and is an essential tool in sustainable agriculture [5,6].

However, depending on the number of variables, the effect of biochar on crop growth is inconsistent [7]. The type of biomass used to generate biochar is a significant factor. Biochars made from various materials exhibit unique properties that can affect their efficiency [8]. In contrast to biochar generated from animal manure, Sarfaraz et al. [9] discovered that biochar made from crop straw is more alkaline, contains more carbon, has a greater cation exchange capacity, emits less CO_2_, and is more effective at improving soil fertility. Rice straw biochar has been demonstrated to be one of the most successful crop straws [10,11]. The temperature at which biochar is created during pyrolysis is another important consideration. In contrast to biochar created at 700 °C, Olszyk et al. [12] showed that biochar produced at 350 °C and 500 °C supports superior crop development. Compared with biochar produced at 400 °C, biochar produced at 600 °C showed an average increase of 12.21% in plant height, kernel number per cob, soil plant analysis development value, cob length, cob girth, grain cob yield, grain yield, test weight, and stover yield. In contrast, Wani et al. [13] found that biochar hindered dry weight buildup when produced at 500 °C and 600 °C, while wheat yield was considerably higher with biochar produced at 300 °C than without biochar application.

Another important consideration is residence time. The properties of biochar can be changed considerably by varying the duration of residence. Longer residence times of biochar result in increased endogenous GA3 content in crops, higher expression levels of genes related to the photoperiod and GA3 pathway, earlier flowering, and increased vegetative and reproductive growth [14]. The ultimate structural properties of biochar, such as pore structure, can vary greatly depending on its raw materials, pyrolysis temperature, and residence time [15,16]. For example, the pore structure of biochar obtained from woody plants is generally more homogeneous, whereas that of biochar derived from herbaceous plants is more heterogeneous [17,18]. Biochar’s aromaticity and structural stability increase with higher pyrolysis temperatures and longer residence times; however, these factors also decrease the structure and specific surface area of biochar pores [19,20]. Therefore, the enhancing effect of biochar on plant growth is directly influenced by its spatial pore structure.

The spatially porous structure of biochar offers soil microorganisms a haven, shielding them from external stresses and improving the quality and accessibility of their habitats [21,22,23]. Numerous methods have been developed to promote microbe-assisted plant development. Instead of preventing harmful microbes from growing, increasing certain beneficial bacteria can actively stimulate plant growth [24]. Microorganisms can improve the plant’s immune system and development, and some rhizosphere bacteria that encourage plant growth can control these aspects [25,26]. Microorganisms can also enhance soil health, which indirectly promotes plant development. Soil microorganisms can improve plant development by strengthening the soil’s nutrient content and structure [27]. Through nitrogen fixation, phosphorus solubilization, and other processes, microorganisms can also supply plants with additional nutrients; the uptake of these nutrients aids plant growth [28]. Aeration, water flow and storage, and nutrient availability are all significantly affected by the quantity and size of pores in biochar, and these factors ultimately shape the microenvironment of microbial communities [29,30].

Therefore, its structure may be a key factor when assessing whether a piece of biochar can sustain autochthonous microbial colonization processes [31,32]. Biochar can interact with soil particles and affect soil microbial populations by altering microhabitats and nutrient cycling [33,34,35]. According to Lu and Zong [36], biochar pores with a 5–20 µm diameter are the most suited for supporting microbial habitation and retaining plant water. Microbial diversity and abundance are positively affected by larger pores (>5 µm) in the soil [37]. According to Wu et al. [38], the porous structure of biochar encourages microbial acclimatization, permits direct interspecies electron transfer, and offers immobilization anchoring points, which eventually impact plant growth.

Despite these insights, the exact processes through which the spatial structure of biochar influences crop development and soil microbial populations remain to be elucidated. Understanding these pathways is critical for maximizing the application of biochar in agriculture. In this context, this study addresses three key objectives: (1) how different pore structures of biochar impact plant development, (2) how the different pore structures influence soil microbial populations, and (3) the underlying mechanisms linking biochar pore structure to both plant growth and microbial populations. To achieve these aims, three types of biochars with varying pore structures were prepared at different temperatures (250 °C, 400 °C, and 550 °C). This study explores how different biochar pore structures impact tobacco growth and soil microbial populations, and the underlying mechanisms. The findings will provide insights into biochar application, contributing to the development of sustainable agricultural practices and global cultivation strategies.

## 2. Results

### 2.1. Differences in the Poly-Spatial Structure of Biochar

**Figure 1:**
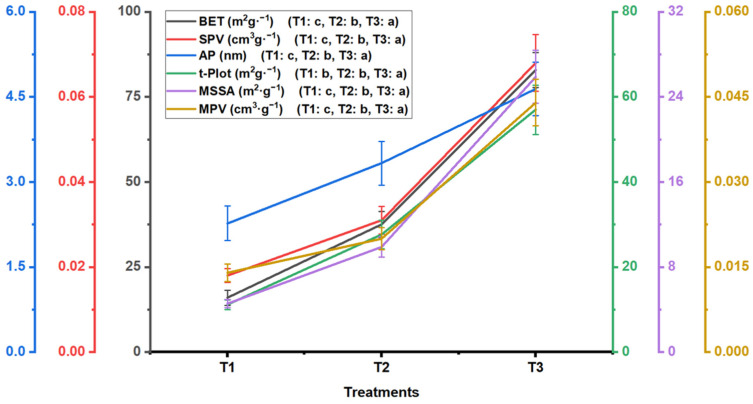
Differences in the poly-spatial structure of biochar among treatments. T1, T2, and T3 represent biochars with poly-spatial structures ranging from the smallest to largest. The error bars indicate the standard deviation. Significant differences are denoted by different lowercase letters in the legend (*p* < 0.05, Tukey’s test). BET, BET specific surface area; SPV, specific pore volume; Ap, average pore size; t.Plot, t.Plot micropore specific surface area; MSSA, mesopore specific surface area; and MPV, mesopore pore volume.

To assess how various biochar pore structures influence the growth of flue-cured tobacco and the associated microbial mechanisms, we produced three types of biochars at different pyrolysis temperatures: 250 °C (T1), 400 °C (T3), and 550 °C (T2). The results showed that the key structural indices, including BET specific surface area (BET), specific pore volume (SPV), average pore size (AP), mesopore specific surface area (MSSA), and mesopore pore volume (MPV), progressively increased from T1 to T3 (Figure 1). Specifically, T3 had significantly higher values for these indices than those in T2 (*p* < 0.05), and T2 had significantly higher values than those in T1 (*p* < 0.05). Additionally, the t.Plot micropore specific surface area (t.Plot) of T3 was significantly higher than that of T1 and T2 (*p* < 0.05), with no significant difference between T1 and T2. On average, the structural characteristics of T1 and T2 were only 30.86% and 49.14% developed, respectively, compared with those of T3. These findings indicate that the biochar produced at the highest pyrolysis temperature (T3) possessed the most developed structure among the treatments. These differences among the treatments may have significant implications for their application, including enhancing plant growth and regulating soil microbial activity.

### 2.2. Effects of Biochar Application with Different Spatial Structures on Plant Growth

To comprehensively determine the effect of different biochar structures on the growth of flue-cured tobacco, plant growth differences among the treatments were evaluated (Table 1). Combining the results observed at 30 and 60 days after transplantation revealed that biochar significantly promoted tobacco growth. Biochar treatment increased the agronomic traits by 25.26% compared with the control (CK). Biochar with larger structures had a more pronounced effect on tobacco growth, with T3 (the biochar with the largest structure) showing the most significant impact. Specifically, the height and largest leaf width in the T3 treatment were significantly greater than those in the other treatments, with T3 reaching 1.22 and 1.11 times the values of T1 and T2, respectively (*p* < 0.05). The impact of different treatments on agronomic traits ultimately contributed to variations in biomass production. T3, which had the largest internal structure, resulted in a significantly higher dry weight compared to the other treatments, with significant differences in the root and total weights (*p* < 0.05). On average, the T3 treatment produced 1.23 and 1.09 times more biomass than that with the T1 and T2 treatments, respectively. These findings suggest that biochar with a larger spatial structure has the most beneficial effect on promoting the growth of the root system and the entire tobacco plant, as evidenced by the enhanced agronomic traits and dry weight accumulation.

### 2.3. Differences in Microbial Communities

**Figure 2:**
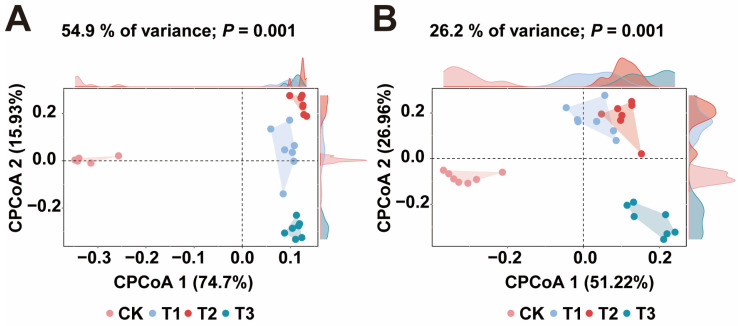
Constrained principal coordinate analysis (CPCoA) illustrating the differences in microbial communities across treatments. CK represents no biochar application, while T1, T2, and T3 represent biochar with poly-spatial structures ranging from the smallest to largest. (**A**) and (**B**) show the CPCoA results for bacterial and fungal communities, respectively.

Building on the finding that biochar with a higher spatial structure promotes better tobacco growth, we further investigated the microbial communities to understand how this spatial structure ultimately influences microbial dynamics to promote tobacco growth. In our bacterial analysis (Table S1) across treatments (including seven replicates), the number of effective tags ranged from 103,842 to 124,954, with an effective ratio between 83.96% and 91.53%. Additional metrics, including Raw PE, Clean PE, Raw Tags, Clean Tags, and Chimera checks, confirmed the reliability of the results. Similarly, in our fungal analysis (Table S2), the number of effective tags ranged from 113,449 to 132,025, with an effective ratio between 93.36% and 96.56%. The reliability of the fungal data was also verified using metrics such as Raw PE, Clean PE, Raw Tags, Clean Tags, and Chimera checks. To assess the variance among treatments, we conducted CPCoA, focusing on the top 500 operational taxonomic units (OTUs) for bacteria and fungi (Figure 2). Our analysis revealed substantial differences in the bacterial and fungal communities among the treatments (PERMANOVA; *p* = 0.001). Specifically, the results (Figure 2A) showed greater variance in bacterial communities than in fungal communities (Figure 2B), indicating that bacteria were more sensitive to variations in biochar spatial structures.

**Figure 3:**
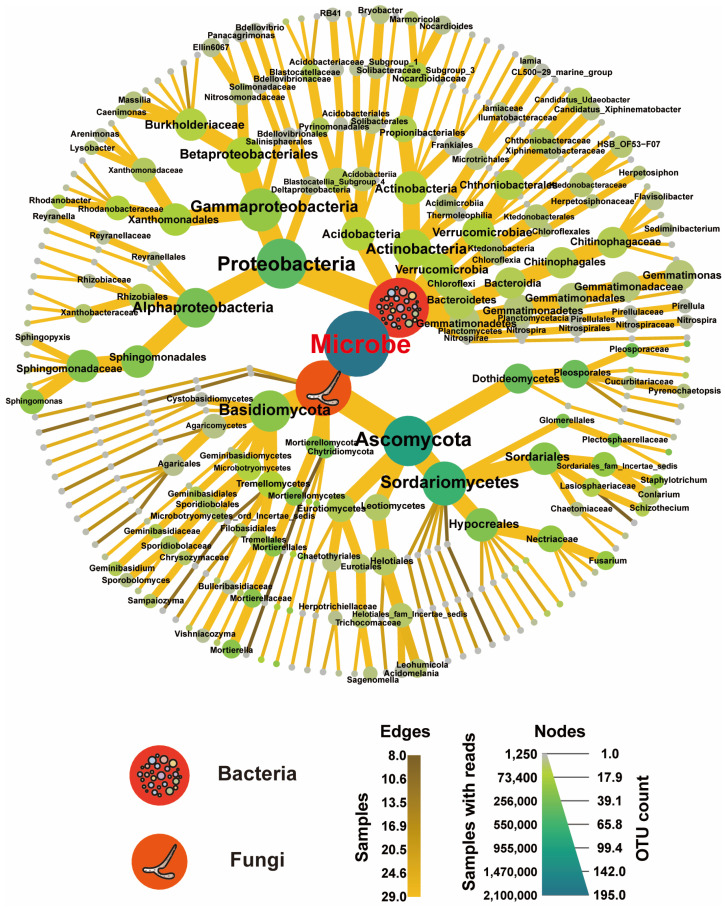
Evolutionary tree plots illustrating the dominant microbes at the domain, kingdom, phylum, class, order, family, genus, and species levels across the four treatments.

We combined the microbial data to understand microbial dynamics further and utilized a heat tree plot to analyze the significant communities among the top 500 bacterial and fungal OTUs (Figure 3). Heat tree plots provide a hierarchical view of microbial community composition, allowing for visualization of the relative abundance and relationships among different taxa. Our analysis revealed that bacterial communities were more abundant than fungal communities. The dominant phyla among the bacterial communities were *Proteobacteria* (58.83%), *Actinobacteria* (10.39%), *Acidobacteria* (8.41%), *Verrucomicrobia* (7.51%), and *Bacteroidetes* (5.20%). The dominant genera were *Sphingomonas* (22.86%), *Rhodanobacter* (5.84%), *Candidatus Udaeobacter* (4.61%), *OLB17* (3.08%), *Sphingobium* (2.88%), *Marmoricola* (2.84%), *HSB OF53-F07* (2.81%), and *Gemmatimonas* (2.61%). The dominant phyla in the fungal communities were *Ascomycota* (63.60%), *Chytridiomycota* (14.59%), *Mortierellomycota* (10.81%), and *Basidiomycota* (10.76%). The dominant genera were *Alternaria* (15.67%), *Plectosphaerella* (13.36%), *Mortierella* (10.81%), *Spizellomyces* (9.89%), *Fusarium* (7.94%), *Staphylotrichum* (7.60%), *Boothiomyces* (4.57%), *Schizothecium* (2.32%), *Geminibasidium* (2.21%), and *Trichoderma* (2.04%).

**Figure 4:**
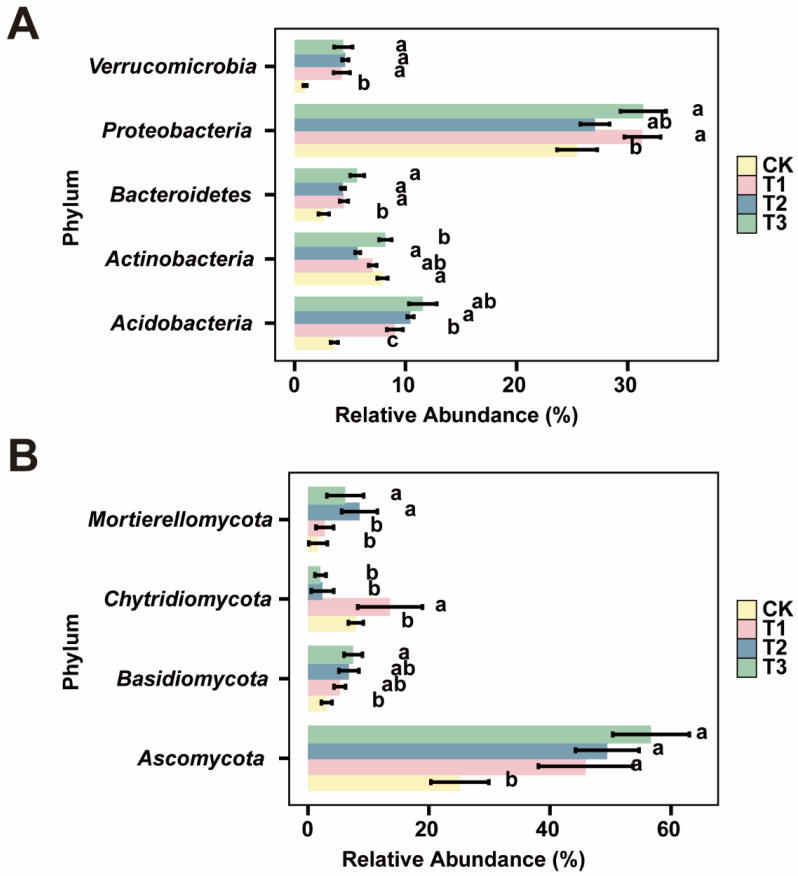
Microbial abundance at the phylum level in the tobacco rhizosphere. Panel (**A**) shows bacterial expression and panel (**B**) shows fungal expression. CK represents no biochar application, while T1, T2, and T3 represent biochar with poly-spatial structures ranging from the smallest to largest. The error bars indicate the standard deviation. Significant differences are indicated by different lowercase letters (*p* < 0.05, Tukey’s test).

We further analyzed the differences in the dominant bacterial and fungal phyla across the different treatments. The results indicated significant differences among the treatments (Figure 4). In the bacterial communities (Figure 4A), the relative abundance (RA) of Verrucomicrobia in the control (CK) was significantly lower than that in the biochar treatments (*p* < 0.05). Additionally, the RA of Proteobacteria in T3 and T1 was significantly higher than in the CK (*p* < 0.05). The RA of Bacteroidetes and Acidobacteria increased with increasing biochar spatial structure. In the fungal communities (Figure 4B), the RA of Basidiomycota and Ascomycota also increased with larger biochar spatial structures, and the RA in the CK was significantly lower than that in T3 (*p* < 0.05). These findings demonstrate that biochar with larger spatial structures significantly alters microbial community composition, promoting specific bacterial and fungal phyla. We further analyzed the differences in the dominant bacterial and fungal phyla across the different treatments. Additionally, there were significant differences in the relative abundances (RAs) of the dominant bacterial and fungal genera (Table 2).

**Figure 5:**
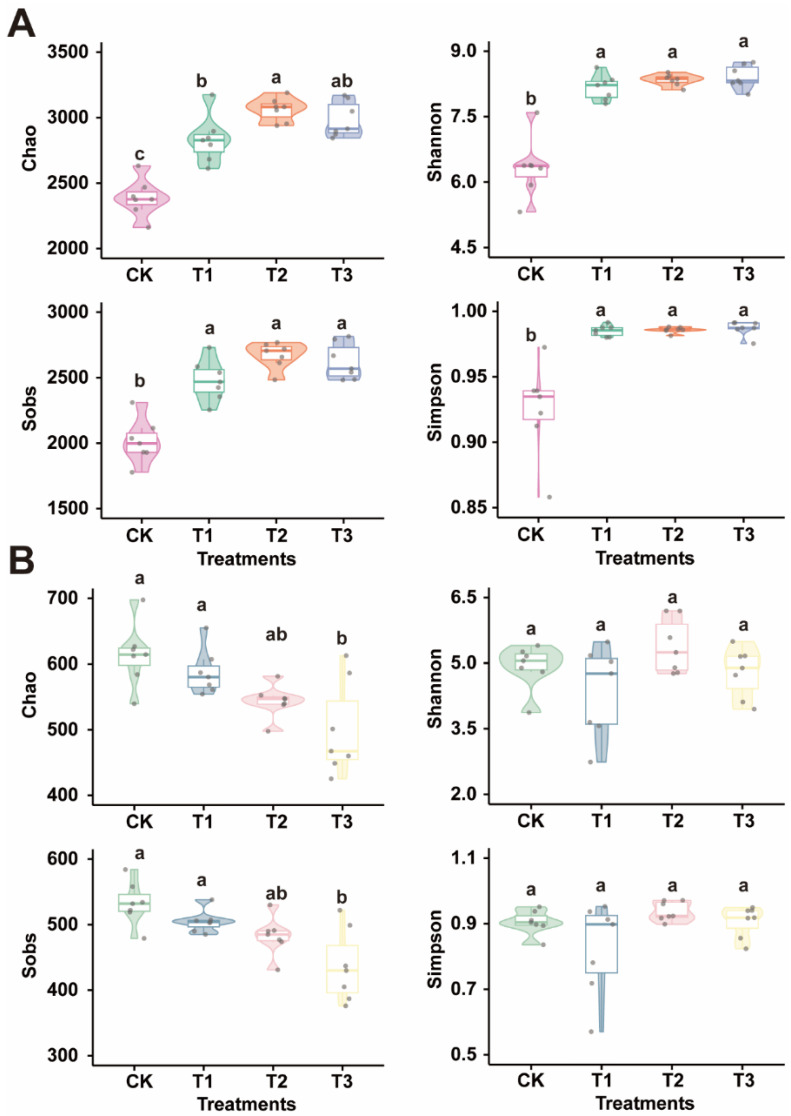
The richness and diversity indices of the soil’s microbial community relative to treatment. The panels (**A**) represent bacteria and panels (**B**) represent fungi. CK represents no biochar application, while T1, T2, and T3 represent biochar with poly-spatial structures ranging from the smallest to largest. Significant differences are denoted by different lowercase letters (*p* < 0.05, Tukey’s test).

In the bacterial communities, the RA of Sphingomonas and Candidatus Udaeobacter in the biochar application treatments was significantly higher than in the CK treatment. Conversely, the RA of Rhodanobacter in the CK treatment was markedly higher than in the biochar treatments (*p* < 0.05). The RA of OLB17 increased with the larger spatial structure in biochar, and the RA in the CK was significantly lower than that in T3 (*p* < 0.05). In the fungal communities, the RA of Alternaria was substantially higher in the biochar treatments than in the CK treatment (*p* < 0.05). Similarly, the RA of Plectosphaerella and Mortierella in the CK was significantly lower than in the biochar treatments (*p* < 0.05). Microbial alpha diversity can provide preliminary insights into environmental health, ecosystem function, and soil fertility. We used box plots to analyze the alpha diversity indices to assess the impact of biochar with different spatial structures on microbial communities (Figure 5). For bacteria (Figure 5A), the Shannon, Sobs, and Simpson indices were significantly lower in the control (CK) than in the biochar treatments (*p* < 0.05). In contrast, the alpha diversity of the fungal communities showed less variation among treatments (Figure 5B). Specifically, the Chao and Sobs indices for T3 were significantly lower than those for the CK, and no significant differences were observed among the other treatments.

### 2.4. Co-Occurrence Networks of Bacterial and Fungal Communities in Relation to the Poly-Spatial Structure of Biochar

**Figure 6:**
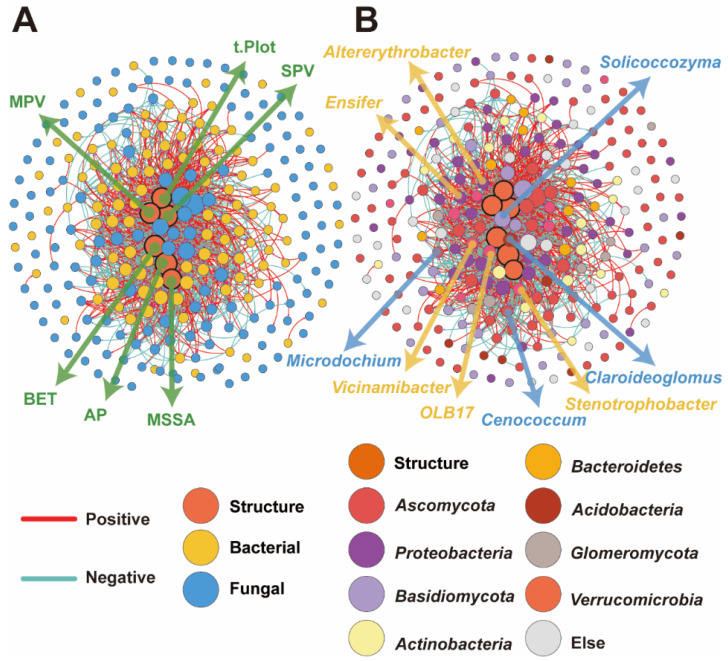
The co-occurrence network of microbial communities under different treatments. Panels (**A**) and (**B**) illustrate the network at the domain and phylum levels, respectively.

The spatial structure of biochar significantly influenced both the bacterial and fungal communities. To further explore these relationships, we conducted a network analysis (Figure 6). Our results show that biochar directly affected the most abundant bacterial and fungal taxa at the domain (Figure 6A) and phylum (Figure 6B) levels. Specifically, specific spatial indices, such as BET, t.Plot, and MSSA, were found to promote microbial abundance. Furthermore, keystone taxa in the bacterial communities included *Stenotrophobacter*, OLB17, *Vicinamibacter*, *Ensifer*, and *Altererythrobacter*. Keystone taxa in the fungal communities included *Glomus*, *Microdochium*, *Solicoccozyma*, and *Cenococcum*.

Regarding network metrics (Table 3), the average degree, network diameter, network density, and clustering coefficient demonstrated close relationships between the biochar spatial structure, bacterial communities, and fungal communities. Furthermore, the connections associated with the AP exhibited the highest closeness and betweenness among the spatial structure indices. BET had the second highest closeness, while MSSA had the second highest betweenness. Conversely, the closeness of the t.Plot and the betweenness of the SPV were lower than those of the other indices. These findings suggest that different spatial indices of biochar have distinct effects on microbial communities, thereby influencing their abundance and interactions within the soil ecosystem. Specifically, in the microbial network analysis for bacteria (Table 4), *Stenotrophobacter* and OLB17, belonging to Acidobacteria, exhibited the highest degree, closeness centrality, and betweenness centrality. In the analysis for fungi, *Claroideoglomus* (Glomeromycota) showed the highest degree and closeness centrality and the second highest betweenness centrality. *Cenococcum*, belonging to Ascomycota, had the lowest closeness centrality but the highest betweenness centrality.

### 2.5. Impact of Poly-Spatial Structure of Biochar and Microbial Communities on Plant Growth

**Figure 7:**
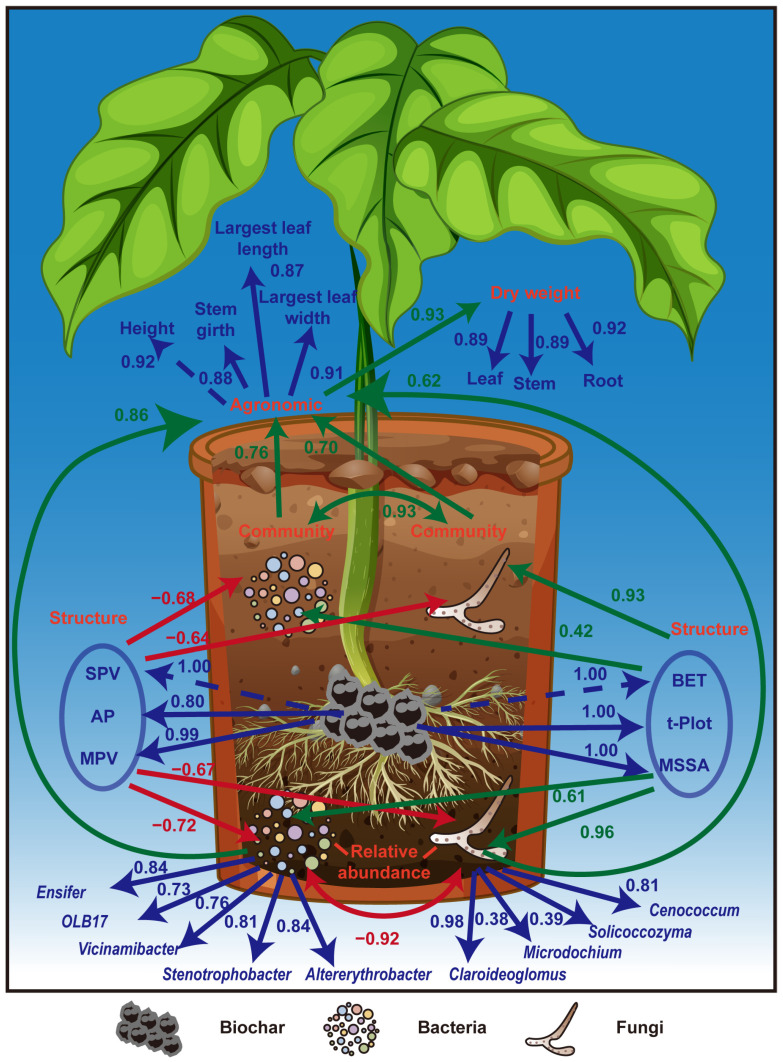
Effects of poly-spatial structure, microbial community composition, and taxa relative abundance on agronomic traits and dry weight, estimated using structural equation modeling. Blue lines represent measurement model. Red lines indicate negative relationships, while green lines indicate positive relationships. Bacterial and fungal communities are represented by their first dominant eigengene, and average clustering coefficients are used to represent taxa. BET; BET specific surface area; SPV, specific pore volume; Ap, average pore size; t.Plot, t.Plot micropore specific surface area; MSSA, mesopore specific surface area; MPV, mesopore pore volume.

Using structural equation modeling (SEM), we further elucidated the relationships among the spatial structure of biochar, community composition, the relative abundance of the key bacteria and fungi in Table 4, agronomic traits, and the dry weight production of tobacco (GFI = 0.86, RMSEA = 0.09, Figure 7). Based on the previous results, the structural indices were divided into two categories: the first category included SPV, AP, and MPV, whereas the second category included BET, t.Plot, and MSSA. The first category adversely affected the microbial community composition and relative abundance, whereas the second category positively influenced the community composition and relative abundance of bacteria and fungi. Furthermore, enhanced microbial community composition and relative abundance promoted plant growth, resulting in improved agronomic traits, ultimately leading to higher dry weight accumulation.

## 3. Discussion

Biochar produced under various conditions, such as different feedstocks, pyrolysis temperatures, and residence times, exhibits distinct effects on plant growth. These effects are primarily attributed to changes in the pore spatial structures, which affect soil microbial populations [15,16]. Our study examined biochar produced at three distinct pyrolysis temperatures and assessed its effects on plant growth, microbial activity, and specific microbiological indices. Structural equation modeling was used to determine how the pore spatial structure of biochar influences plant growth. Our findings show that the pyrolysis temperature substantially influences the pore spatial structure of the biochar, consistent with previous research [14,15,16]. Biochar produced at 400 °C (T3) had the biggest pore spatial structure, with volumes 3.24 times and 2.04 times greater than those produced at 250 °C (T1) and 550 °C (T2), respectively. According to Zhang et al. [39] and Břendová et al. [40], increasing the pyrolysis temperature increases the total and micropore volumes of biochar through cellulose breakdown and aromatic ring fusion. However, excessively high temperatures may also cause the porous structure to melt, resulting in a decreased spatial pore structure.

According to our analysis, treatment with biochar produced at 400 °C significantly increased plant growth and biomass accumulation (T3). Compared to plants cultivated with biochar produced at 250 °C (T1) and 550 °C (T2), plants grown with this biochar showed superior growth, with growth improvements of 1.49-, 1.64, and 1.80 times, respectively, relative to that with the control (CK). This validates earlier studies suggesting that the best pyrolysis temperatures for biomass accumulation and plant growth are moderate [13]. Subsequent examination revealed notable discrepancies in the relative abundance of microorganisms at the phylum and genus levels among the various biochar applications. Biochar treatments significantly affected the α-diversity and abundance of bacteria and fungi, such as Ascomycota, Basidiomycota, and Mortierellomycota. Plant production, soil health, and nutrient cycling depend on these microbial communities [41,42]. In addition to variation in the changes in biochar structure among treatments, our research identified important microbial taxa at the genus level, including *Stenotrophobacter*, *OLB17*, *Vicinamibacter*, *Ensifer*, *Altererythrobacter*, *Claroideoglomus*, *Microdochium*, *Solicoccozyma*, and *Cenococcum*. These taxa also significantly affect plant growth by promoting root development, nutrient cycling, and stress resistance; for example, *Stenotrophobacter*, a key taxa influenced by the poly-spatial structure of biochar, can increase plant biomass and biofilm formation [43]. *OLB17* may aid in K utilization, optimize soil health, and enhance grapevine resilience [44]. *Vicinamibacteria* improves metabolic functions, reducing Cd enrichment in cotton and promoting growth [45]. *Ensifer* fixes nitrogen in symbiosis with plants [46]. *Altererythrobacter* dissolves potassium compounds, boosting crop yield [47]. *Claroideoglomus* enhances antioxidant enzyme activities, improving crop yield and quality [48]. *Solicoccozyma* stimulates root growth by releasing auxins and lipids [49]. *Cenococcum*, an *ectomycorrhizal* fungus, enhances plant nutrient absorption and stress resistance [50]. While these microbes are beneficial for plant growth, *Microdochium* is not. Furthermore, microbial community composition is essential for soil carbon and nitrogen dynamics, which affect nutrient availability and enzyme activity, impacting crop growth and plant production [51,52,53,54].

We examined the relationship between soil bacteria and biochar pore structure by combining the data from all treatments [55]. Our findings showed that microbial abundance and pore spatial structure were closely related. SPV, AP, and MPV negatively affected microbial abundance, whereas BET, t.Plot, and MSSA positively influenced microbial abundance. The abundances of nitrogen-fixing bacteria (nifH gene), nitrifying bacteria (amoA gene), and denitrifying bacteria (nirK, nirS, and nosZ genes) were increased by biochar with larger specific surface areas (BET, t.Plot, and MSSA). The microbiological abundance in the soil and biochar surface was positively correlated with the abundance of these functional genes [56]. Furthermore, the greater specific surface area of biochar increases the number of bacteria that reduce N_2_O (nosZ gene), which in turn lowers N_2_O emissions [57]. In bacteria on the surface of biochar, metagenomic analysis revealed a gene cluster with NH_4_^+^, NO_3_^−^, and NO_2_ transport-related genes, as well as a urease operon with urea transport genes [58]. This implies that the increased BET, t.Plot, and MSSA could encourage nitrogen transformation and plant development. Biochar with a large specific surface area helps retain water and nutrients, offers abundant resources for microbial activity, and creates a suitable habitat for soil microorganisms [59,60].

In contrast, microbial taxa’s relative abundance may decrease, and microbial communities’ composition may change with increased pore volumes and sizes (SPV, AP, and MPV). This could be because of the challenges bacteria and fungi face when utilizing and breaking down nutrients on the biochar surface [33]. The ability of microorganisms to adhere to the surface can be reduced by increasing the pore volume [61,62]. Furthermore, excessively large holes may adversely affect anaerobic digestion processes, thus lowering digestion efficiency and microbial activity [63]. The impact of biochar pore spatial structure on plant growth was clarified using structural equation modeling. Higher t.Plot, MSSA, and BET improved the growth conditions for microorganisms, increasing their relative abundance and maximizing community composition. In contrast, higher plant dry weight is ultimately achieved by improving agronomic features and adversely affecting the soil microbial ecosystem, owing to increased SPV, AP, and MPV.

## 4. Materials and Methods

### 4.1. Biochar Preparation and Soil Characteristics

Tobacco stalks from the “Yunyan 87” cultivar (cut to 2–3 cm) were pyrolyzed at 250 °C, 400 °C, and 550 °C to produce biochar, with all other conditions remaining constant. The soil used in this experiment was red clay, derived from granite and gneiss, collected from Longyang District, Baoshan City, Yunnan Province, China (E98°43′–99°26′, N24°46′–25°38′). This pathogen-free red clay soil has a soil profile consisting of an organic matter-rich surface layer, a middle clay layer, and a bottom mineral layer. The soil properties were as follows: pH 5.63, 8.49 g·kg^−1^ of organic matter, 120.13 mg·kg^−1^ of available potassium, 2.32 mg·kg^−1^ of available phosphorus, and 61.86 mg·kg^−1^ of available nitrogen.

### 4.2. Biochar Characterization

Microscopic Morphology: Ten milligrams of biochar were mounted, gold sputter-coated, and examined under a scanning electron microscope (FlexSE1000, Fukuoka, Japan) to observe its morphology; seven replicates were analyzed [64]. Specific Surface Area and Pore Analysis: Specific surface area and pore size distribution were determined using a Quadrasorb Si automated surface area and micropore analyzer (Boynton Beach, FL, USA) with seven replicates [65].

### 4.3. Experimental Design

The experiment was conducted at a greenhouse in Longyang District, Baoshan City, Yunnan Province, China, from April to June 2021. A completely randomized design was used, with three biochar treatments based on the pyrolysis temperature: 250 °C (T1), 400 °C (T3), and 550 °C (T2). The treatments resulted in varying pore structures, ranging from small to large. Each treatment included 60 pots, with 20 pots replicated thrice, thus totaling 180 pots. Tobacco cultivar “Yunyan 87” seedlings (45 days old) were transplanted into 35 × 35 cm pots containing 10 kg of soil mixed with 200 g of biochar and 20 g of base fertilizer per pot [66].

### 4.4. Agronomic Traits and Dry Weight Measurement

Plant growth indicators were measured in three selected tobacco plants from each treatment at 30 and 60 days after planting [67,68]. Plant Height: Plant height was measured from the soil surface to the topmost point of the stem. Stem Girth: Stem girth was measured at the midpoint of the stem circumference. Largest Leaf Length and Width: The largest leaf was selected, and its length (from leaf base to tip) and width (at the widest point) were measured. The leaf area was calculated using a conversion factor of 0.63. Dry Weight: Plants were divided into roots, stems, and leaves. Three plants per treatment were sampled, rinsed to remove soil, and dried at 105 °C for 30 min, followed by 75 °C for 72 h [69]. The dry weight of each sample was then measured.

### 4.5. Soil Sampling and DNA Extraction

Soil samples were collected 60 days after transplanting, and DNA was extracted from 0.5 g of soil using the Mo Bio^®TM^ DNA isolation kit (Mo Bio Laboratories, Inc., Carlsbad, CA, USA) [70]. DNA integrity was confirmed by 1% agarose gel electrophoresis, and DNA purity and concentration were assessed using a mini-drop spectrophotometer. Bacterial communities were analyzed by amplifying the V3-V4 region of the 16S rDNA gene using the primers 341f and 806r [71]. Fungal communities were assessed using the primers ITS1_F_Kyo2 and ITS86r to amplify the ITS1 region [72]. The amplified products were sequenced on an Illumina MiSeq platform by Guangzhou Gideo Biotechnology Co., Ltd. (Guangzhou, China). After sequencing, raw reads were filtered, assembled, and merged into tags. Effective tags were clustered into operational taxonomic units (OTUs) using the UPARSE algorithm, and chimeric tags were removed using the UCHIME algorithm [73]. OTU abundance was calculated based on the effective tags, and taxonomic annotation was performed using the RDP classifier Bayesian algorithm against the Silva (bacteria) and UNITE (fungi) databases [74].

### 4.6. Bioinformatics and Statistical Analyses

Data processing was performed using Microsoft Excel 365 with graphical representation in Origin 2022 (Northampton, MA, USA), and R 4.3.3. Tukey’s multiple range test (*p* < 0.05) was performed using SPSS (version 23.0; Armonk, NY, USA). Constrained principal coordinate analysis (CPCoA) plots were generated using the “vegan” package in R 4.3.3 [75], and the differences in the composition of microbial communities were assessed by PERMANOVA using “Adonis” in R 4.3.3. The evolutionary tree plots were generated using the “ggClusterNet” package in R 4.3.3 [76], bar and box plots were created using the “ggplot2” package in R 4.3.3 [77], and network analysis plots were generated using the “ggcluster” package in R 4.3.3 [55]. All figures were refined and compiled using Adobe Illustrator version 2019 (San Jose, CA, USA).

## 5. Conclusions

In summary, the surface aspects of biochar pore spatial structure are critical for stimulating plant growth, whereas volume factors can impede it. Biochar produced at 400 °C was found to have the largest and most advantageous pore structure, leading to enhanced tobacco growth and biomass accumulation. Biochar with larger specific surface areas (BET, t.Plot, and MSSA) positively influenced the abundance of key microbial taxa (*Stenotrophobacter*, *OLB17*, *Vicinamibacter*, *Ensifer*, *Altererythrobacter*, *Claroideoglomus*, *Microdochium*, *Solicoccozyma*, and *Cenococcum*) and community composition, thereby promoting plant growth. Conversely, larger pore volumes (SPV, AP, and MPV) hindered microbial activity and altered community composition, negatively affecting plant growth. The structural equation model explained the mechanisms by which the pore structure of biochar influenced plant growth. However, future research integrating metagenomic analyses is essential to elucidate the species-level mechanisms underlying these interactions, ultimately refining our understanding of the role of biochar in sustainable agriculture and soil management and thereby enhancing agricultural productivity.

## Figures and Tables

**Table 1:** Effects of biochar application with different spatial structures on agronomic traits and dry weight accumulation at 30 and 60 days post-transplantation.

Days After Transplantation	Treatments	Height (cm)	Stem Girth (cm)	Largest Leaf Length (cm)	Largest Leaf Width (cm)	Day Weight (g)			
						Root	Stem	Leaf	In Total
30 d	CK	26.08c	0.87c	33.05c	11.84c	3.18c	12.27c	27.73b	43.18b
	T1	29.81b	0.97bc	34.62bc	12.74bc	3.48c	12.97bc	30.10b	46.55b
	T2	33.21b	1.07ab	36.93ab	13.35b	3.94b	14.57ab	34.40a	52.91b
	T3	37.64a	1.22a	40.45a	15.13a	4.36a	15.82a	36.70a	56.88a
60 d	CK	50.65c	1.15c	51.38c	15.64b	5.26c	29.57c	50.35c	85.18c
	T1	55.96c	1.23bc	57.74b	17.57b	5.56c	31.54c	57.54b	94.64b
	T2	62.35b	1.32ab	61.59 ab	19.75a	6.20b	35.15b	64.67a	106.02b
	T3	68.29a	1.39a	66.29a	21.06a	6.91a	39.16a	68.98a	115.05a

Here, CK represents no biochar application, while T1, T2, and T3 represent biochar with poly-spatial structures ranging from the smallest to largest. Significant differences are denoted by different lowercase letters (*p* < 0.05, Tukey’s test).

**Table 2:** Microbial abundance at the genus level in the tobacco rhizosphere.

Bacterial Abundance					
Treatments	*Sphingomonas*	*Rhodanobacter*	*Candidatus*_*Udaeobacter*	*OLB17*	*Sphingobium*
CK	1.58b	5.67a	0.29b	0.02c	0.48b
T1	8.82a	0.42b	1.78a	0.75b	0.99ab
T2	6.85a	0.14b	2.18a	1.28ab	0.51b
T3	9.32a	0.26b	1.54a	1.38a	1.49a
**Fungal Abundance**					
**Treatments**	***Alternaria***	***Plectosphaerella***	***Mortierella***	***Spizellomyces***	***Fusarium***
CK	1.47b	0.39b	1.68b	0.17b	0.29b
T1	3.93b	11.89a	2.79a	13.34a	9.90a
T2	2.27b	6.09a	8.53a	2.35b	1.08ab
T3	19.60a	5.42a	6.17a	1.09b	2.17a

Here, CK represents no biochar application, while T1, T2, and T3 represent biochar with poly-spatial structures ranging from the smallest to largest. Significant differences are denoted by different lowercase letters (*p* < 0.05, Tukey’s test).

**Table 3:** Summary of keystone taxa network metrics across different treatments.

Index	Nodes	Positive	Negative	Closeness	Betweenness	Average Degree	Network Diameter	Network Density	Clustering Coefficient
BET	1	46	25	0.599	977.353	14.117	6.000	0.035	0.233
SPV	1	24	46	0.594	773.195				
AP	1	25	52	0.610	1454.454				
t.Plot	1	44	21	0.580	920.012				
MSSA	1	42	22	0.569	1145.989				
MPV	1	24	43	0.586	823.236				
Microbe	256	338	455	0.444	134.141				

BET, BET specific surface area; SPV, specific pore volume; Ap, average pore size; t.Plot, t.Plot micropore specific surface area; MSSA, mesopore specific surface area; MPV, mesopore pore volume.

**Table 4:** Network metrics of keystone bacterial and fungal taxa.

Domain	Phylum	Genus	Degree	Closeness Centrality	Betweenness Centrality
Bacterial	*Acidobacteria*	*Stenotrophobacter*	25	0.492	179.164
	*Acidobacteria*	*OLB17*	25	0.495	179.313
	*Acidobacteria*	*Vicinamibacter*	22	0.488	107.522
	*Proteobacteria*	*Ensifer*	21	0.480	37.320
	*Proteobacteria*	*Altererythrobacter*	20	0.478	34.499
Fungi	*Glomeromycota*	*Claroideoglomus*	34	0.495	77.160
	*Ascomycota*	*Microdochium*	17	0.463	28.869
	*Basidiomycota*	*Solicoccozyma*	24	0.469	43.896
	*Ascomycota*	*Cenococcum*	22	0.449	247.311

## Data Availability

Data are contained in the Supplementary Materials of the article and further inquiries can be directed to the corresponding author.

## Supplementary Materials

The following supporting information can be downloaded at: https://www.mdpi.com/article/10.3390/plants13212952/s1. Table S1. Sequencing data processing for variable regions of bacteria (16S; V3-V4). Table S2. Sequencing data processing for variable regions of fungi (ITS1_plant).
