# Supplementary material for: Exploring the Relationship Between Biochar Pore Structure and Microbial Community Composition in Promoting Tobacco Growth
Source: Plants (Basel). 2024 Oct 22;13(21):2952. doi: 10.3390/plants13212952 (PMC11548322; doi:10.3390/plants13212952)
Supplement: Supplementary file 1 [file plants-13-02952-s001.zip › plants-3230975-supplementary.pdf]

Table S1. Sequencing data processing for variable regions of bacteria (16S; V3-V4).

Sample Name	Raw PE	Clean PE	Raw Tags	Clean Tags	Chimera	Effective Tags	Effective Ratio (%)
CK_1	130141	129994	128041	127446	15063	112383	86.35
CK_2	123754	123614	121888	121314	13716	107598	86.95
CK_3	135297	135119	133075	132419	13003	119416	88.26
CK_4	126786	126637	124657	123980	11617	112363	88.62
CK_5	127539	127373	125471	124800	11051	113749	89.19
CK_6	128602	128466	126584	125998	11854	114144	88.76
CK_7	133024	132861	130895	130210	13775	116435	87.53
T1_1	122229	122098	120368	119885	15178	104707	85.66
T1_2	122002	121854	119856	119202	14542	104660	85.79
T1_3	130646	130499	128341	127645	16880	110765	84.78
T1_4	136104	135943	133664	132956	15909	117047	86.00
T1_5	128050	127907	125554	124857	17344	107513	83.96
T1_6	130324	130177	128172	127552	17961	109591	84.09
T1_7	132867	132696	130375	129563	17061	112502	84.67
T2_1	120041	119923	117860	117224	13256	103968	86.61
T2_2	133077	132908	130478	129703	15268	114435	85.99
T2_3	131730	131591	129523	128928	14483	114445	86.88
T2_4	124741	124596	122569	122014	14316	107698	86.34
T2_5	123076	122932	120688	119943	13751	106192	86.28
T2_6	132703	132533	130179	129453	14872	114581	86.34
T2_7	137384	137220	134800	134034	16397	117637	85.63
T3_1	124538	124383	122330	121634	15652	105982	85.10
T3_2	123082	122913	120784	120108	16266	103842	84.37
T3_3	121777	121651	119798	119195	7728	111467	91.53
T3_4	137509	137336	134885	134241	9878	124363	90.44
T3_5	136640	136476	134161	133481	10358	123123	90.11
T3_6	124544	124384	122280	121630	10588	111042	89.16
T3_7	136791	136631	134642	134082	9128	124954	91.35

Table S2. Sequencing data processing for variable regions of fungi (ITS1_plant).

Sample Name	Raw PE	Clean PE	Raw Tags	Clean Tags	Chimera	Effective Tags	Effective Ratio (%)
CK_1	127586	127519	123402	122785	1029	121756	95.43
CK_2	129527	129424	123777	123025	757	122268	94.40
CK_3	131505	131410	125247	124452	341	124111	94.38
CK_4	120418	120332	115825	115033	330	114703	95.25
CK_5	125161	125073	118403	117597	464	117133	93.59
CK_6	125896	125811	122578	121759	554	121205	96.27
CK_7	136494	136442	131739	131209	497	130712	95.76
T1_1	131316	131231	123819	123089	221	122868	93.57
T1_2	131402	131313	125556	124890	325	124565	94.80
T1_3	121423	121348	117135	116374	624	115750	95.33
T1_4	124655	124558	121064	120351	328	120023	96.28
T1_5	134048	133980	128186	127496	646	126850	94.63
T1_6	131813	131739	128259	127742	457	127285	96.56
T1_7	131682	131620	127359	126573	248	126325	95.93
T2_1	137190	137119	132909	132444	419	132025	96.24
T2_2	120965	120898	117422	116916	600	116316	96.16
T2_3	120289	120208	116664	116138	647	115491	96.01
T2_4	132767	132664	126554	125989	1577	124412	93.71
T2_5	135623	135485	127978	127375	378	126997	93.64
T2_6	120357	120270	114308	113756	307	113449	94.26
T2_7	136117	136014	128937	128456	1375	127081	93.36
T3_1	126878	126766	120105	119386	776	118610	93.48
T3_2	124188	124099	119652	118929	404	118525	95.44
T3_3	120885	120817	115625	115135	682	114453	94.68
T3_4	133301	133236	127193	126650	502	126148	94.63
T3_5	128239	128180	124378	123742	412	123330	96.17
T3_6	132760	132690	128183	127594	1320	126274	95.11
T3_7	129467	129409	125292	124671	677	123994	95.77
